# Intravenous human immunoglobulin utilization patterns and cost analysis in a Malaysian tertiary referral hospital

**DOI:** 10.1186/s40545-022-00430-2

**Published:** 2022-04-26

**Authors:** Shea Jiun Choo, Chun Zheng Ng, Yi Jing Ong, Kamariah Shamsinar Kamarul Baharin, Chee Tao Chang

**Affiliations:** 1 Pharmacy Department, Hospital Taiping, Ministry of Health Malaysia, 34000 Taiping, Malaysia; 2 Clinical Research Centre, Hospital Raja Permaisuri Bainun, Ministry of Health Malaysia, 30450 Ipoh, Malaysia

**Keywords:** Immunoglobulins, Drug utilization, Cost analysis, Hospitals, Malaysia

## Abstract

**Introduction:**

While intravenous human immunoglobulin therapy is potentially lifesaving for rare diseases, the significant costs associated with its usage warrant due attention. This study evaluated the costs and prescribing patterns of IVIg.

**Methods:**

This was a retrospective analysis of medical records in a tertiary hospital. The evidence category IIA and below, as well as strength of recommendations level B and below were classified as lower evidence category and lower strength of recommendation, respectively. Patients’ demographic data, indications, dosing regimen, physician specialty were retrieved from medical records, while the cost was derived based on total prescribed doses.

**Results:**

Out of 78 patients, more than half of the patients were prescribed with off-label IVIg based on MOHM Formulary (52, 66.7%), FDA indications (52, 66.7%) and EMA indications (46, 59.0%). 37 (47.4%) cases used IVIg for indications with lower evidence category and 52 (66.7%) cases with lower strength of recommendation. The total cost of IVIg use within the 2-year period was RM 695,426.36, with RM267,993.40 (38.5%) spent for indications with lower evidence category. Immunoglobulin use in rheumatology and neurology cases were associated with lower evidence category (*p* < 0.001).

**Conclusions:**

A high proportion of off-label immunoglobulin use was observed. A timely update of prescribing policy, standardization of prescribing guidelines may promote appropriate immunoglobulin prescribing and justify expenses.

**Supplementary Information:**

The online version contains supplementary material available at 10.1186/s40545-022-00430-2.

## Background

Intravenous immunoglobulin (IVIg) is a blood product derived from human donor blood that contains a mixture of antibodies (immunoglobulins) in the form of preparation for intravenous injection. IVIg was initially used as a treatment for immune deficiencies, and later for various autoimmune and inflammatory disorders. Currently, there are ten approved indications for the use of IVIg, as established by the United States Food and Drug Administration (FDA) and European Medicines Agency (EMA) [1, 2].

Although manufacturing steps have been implemented to enhance the purity of the Immunoglobulin G molecules [3], its administration can lead to adverse events and potential fatal consequences. While IVIg therapy can be lifesaving for specific rare diseases, the significant costs associated with its usage warrant due attention. The total cost of IVIg usage may vary depending on several factors, such as the indication, duration of treatment and the patient’s body weight. In a utilisation review carried out in a tertiary care hospital in United Arab Emirates, the estimated annual cost of IVIg was US$1.25 million, in which US$0.7 million was associated with off-label indications [4]. In the USA, the average cost per IVIg infusion for chronic inflammatory demyelinating polyneuropathy was US$9720 [5].

In year 2000, Chen and colleagues reported that 52% of the study subjects from twelve institutions were treated with IVIg for off-label indications. Notably, no positive clinical outcome was observed among 12% in the label group and 20% in the off-label group [6]. Even though off-label indications were supported by limited evidence, there were over 150 off-label indications practiced among the prescribers, incurring substantial cost to the healthcare system [7].

Within the local context, two brands of IVIg with the proprietary name of Intragram^®^ (6 g of human protein IgG in 100 ml) and Flebogamma^®^ (10 g of human protein IgG in 100 mL). were listed in the formulary. Currently, more than three-fourth of the local IVIg was supplied by the National Blood Centre, with a subsidized cost of RM 200 per vial, and an actual cost of RM 500 per vial.

Prescribing of IVIg is primarily guided by the Ministry of Health Drug Formulary, but reference were rarely made to FDA, EMA, American Academy of Allergy, Asthma, and Immunology (AAAAI) evidence category and strength of recommendations for off-label use. There was a paucity of studies on the IVIg prescribing patterns and costs across different groups of patients and prescribers in Malaysia. Hence, this study aimed to evaluate the costs and prescribing patterns of IVIg in term of indications, off-label use, evidence level and strength of recommendation based on established guidelines.

## Methods

This was a retrospective study conducted by the Pharmacy Department of Hospital Taiping, Malaysia. In this study, consecutive sampling method was employed. All neonatal, paediatric and adult patients who received IVIg treatment between 1st January 2019 and 31st December 2020 were included. Patients with untraceable medical records were excluded.

From the IVIg registry, a total of 91 patients prescribed with IVIg within the study period were identified. Patients’ demographic data, indications and dosing regimen of IVIg as well as physician specialty who prescribed IVIg were collected from the medical record office. The cost of IVIg was obtained from the supplier.

The indications of IVIg were compared to the indications approved by MOHM [8], FDA [1] and EMA [2]. Off-label indication was defined as IVIg use in any clinical conditions other than those approved by these three authorities. The evidence category and strength of recommendations for each indication were labelled according to the 2017 Update on the Use of Immunoglobulin in Human Disease: A review of evidence (Additional file 1: Tables S1 and S2) [9]. The evidence category IIA and below, as well as strength of recommendations level B and below were classified as lower evidence category and lower strength of recommendation, respectively.

SPSS version 26 (SPSS®, Chicago, IL) was used to perform all the statistical analyses. The cost and utilization of IVIg in accordance to evidence category and strength of recommendation were descriptively analyzed. The Chi Square test was used to determine the association between off-label use, indications and physician specialty with the evidence category and strength of recommendation. All statistical tests were two-tailed. A *p* value of <0.05 denoted statistical significance.

This study was registered in the National Medical Research Registry (NMRR-21-853-59575) and ethical approval for this study was obtained from the Medical Research and Ethics Committee (MREC), Ministry of Health Malaysia

## Results

Initially, 91 patients were included in this study. Only 78 patients were eligible for analysis as 13 patients had untraceable medical records. Intragram^®^ was used in 76 cases, while Flebogamma^®^ in two cases. Majority were female (*n* = 57, 57.7%) and adult (*n* = 52, 66.7%). The median treatment duration was 2 days and the median total IVIg dose administered per patient was 54.0 gram. More than half of the patients were prescribed with off-label IVIg based on MOHM Formulary (52, 66.7%), FDA indications (52, 66.7%) and EMA indications (46, 59.0%). There were 37 (47.4%) cases which used IVIg for indications with lower evidence category and 52 (66.7%) cases with lower strength of recommendation (Table 1).

**Table 1:** Characteristics of patients and prescribing patterns (*n* = 78)

Characteristics	Frequency	Percentage
Gender		
Male	33	42.3
Female	45	57.7
Age		
Adults	52.0	66.7
Pediatrics	26.0	33.3
Duration of treatment (days, Median, IQR)	2 (3)	–
Total dose of IVIg (gram, median, IQR)	54.0 (91.5)	–
Compliance to MOHM Formulary		
Yes	26	33.3
No	52	66.7
Compliance to FDA indications		
Yes	26	33.3
No	52	66.7
Compliance to EMA indications		
Yes	32	41.0
No	46	59.0
Physician specialty		
Pediatrics	26	33.3
Neurology	20	25.6
Hematology	14	17.9
Rheumatology	12	15.4
Medical	2	2.6
Intensive care units	2	2.6
Dermatology	2	2.6
Evidence category		
Ia	23	29.5
Ib	18	23.1
IIa	2	2.6
IIb	0	0
III	23	29.5
IV	0	0
No established evidence	12	15.4
Strength of recommendation		
A	26	33.3
B	17	21.8
C	2	2.6
D	21	26.9
No established evidence	12	15.4

^a^Adults (median age years±IQR: 40.0±40.0), ^b^Pediatrics (median age days±IQR: 76.0±317.4)

Chi-square test was performed and it was found that IVIg use in systemic lupus erythematosus (SLE) (9, 11.5%) and neonatal jaundice secondary to isoimmunehaemolytic disease (IHD) (9, 11.5%) were associated with lower evidence category (*p *< 0.001). Besides, IVIg prescribing by rheumatology (12, 15.4%) and neurology (20, 25.6%) were associated with lower evidence category (*p *< 0.001) as well as lower strength of recommendation (*p *< 0.001).

**Fig. 1:**
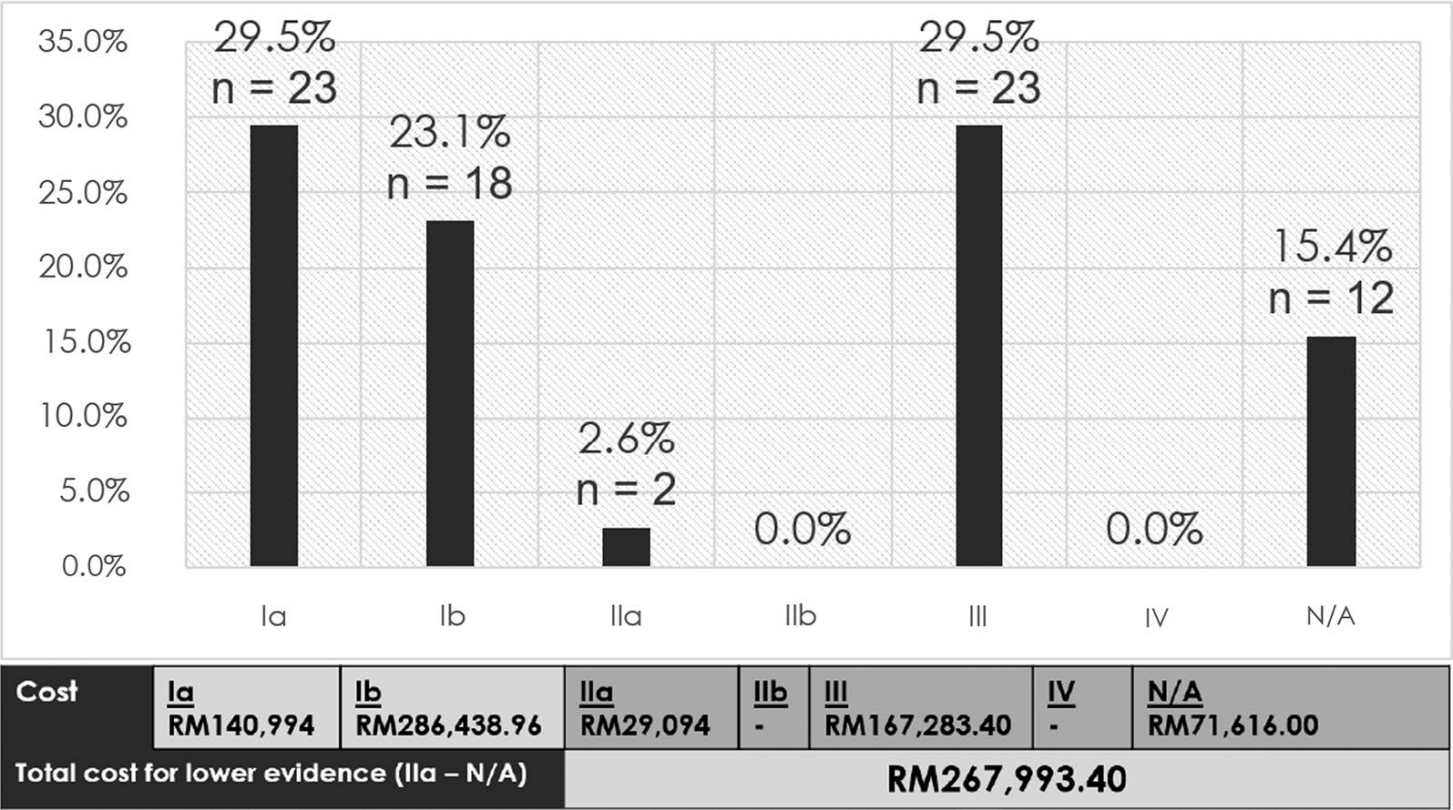
Total cost based on evidence category

The total cost of IVIg use within the 2-year period was RM 695,426.36. In terms of physician specialty, neurology spent the most (RM 356,189.96), followed by rheumatology (RM 142,665.40), hematology (RM 88,401.00), pediatrics (RM 36,927.00), dermatology (RM 29,094.00), medical (RM 23,126.00) and intensive care unit (ICU) (RM 19,023.00). Based on indications, myasthenia gravis reported the highest cost (RM 121,598.00, *n *= 9), followed by SLE (RM 112,825.40, *n *= 9), severe refractory idiopathic thrombocytopenic purpura (ITP) (RM 88,774.00, *n *= 10) and Guillain–Barre syndrome (RM 88,401.00, *n *= 6) (Table 2). The total cost spent on indications with lower evidence category was RM267,993.40, whereby RM71,616.00 was spent on diagnoses with no established evidence support (Fig. 1 and Additional file 1: Table S3).

**Table 2:** Total cost of IVIg use on indications based on evidence category

Indications	Evidence category^a^	N	Mean, Standard deviation/Median, IQR (RM)	95% Confidence interval for mean (RM)	Total cost (RM)
Severe refractory ITP	Ia	10	8877.40 (SD:3598.37)	6303.28–11,451.52	88,774.00
Kawasaki disease	Ia	8	2564.38 (SD: 965.23)	1757.42–3371.33	20,515.00
Immune thrombocytopenia or ITP	Ia	4	1492.00 (IQR: 1025.75)	508.46–2289.04	5595.00
Chronic inflammatory demyelinating polyneuropathy^#^	Ia	1	–	–	26,110.00
Myasthenia gravis	Ib	9	11,936.00 (IQR: 4849.00)	6236.65–20,785.13	121,598.00
Guillain–Barre syndrome	Ib	6	14,733.50 (SD: 6472.38)	7941.15–21,525.85	88,401.00
Chronic lymphatic leukemia not responding to conventional therapy*	Ib	2	1305.50 (SD: 263.75)	–	2611.00
Multifocal motor neuropathy^#^	Ib	1	–	–	73,828.96
Toxic epidermal necrolysis	IIa	2	14,547.00 (SD: 4747.51)	–	29,094.00
Neonatal jaundice secondary to isoimmunehaemolytic disease of newborn	III	9	373.00 (SD: 0.00)	373.00–373.00	3357.00
Systemic lupus erythematous	III	9	12,536.16 (SD: 7679.47)	6633.19–18,439.12	112,825.40
Autoimmune encephalitis*	III	2	11,190.00 (SD: 5275.02)	–	22,380.00
Limbic encephalitis*	III	2	13,987.50 (SD: 1318.75)	–	27,975.00
Neonatal alloimmune thrombocytopenia^#^	III	1	–	–	746.00
Other indications with no established evidence^b^	Not established	12	5968.00 (SD:5224.42)	2648.56–9287.44	71,616.00
Total		78			695,426.36

^a^Based on AAAAI classifications (Perez et al.) ^b^Data available in Additional file 1: Table S3 ^#^Insufficient case to generate mean (SD) and confidence interval. *Insufficient case to generate confidence interval

## Discussion

In this study, more than half of the IVIg treatment were prescribed for off-label indications. A study on IVIg prescribing patterns in four teaching hospitals in Toronto revealed that over 80% of the patients received IVIg for indications supported by published recommendations [10]. On the other hand, 47.5% of the cases in this study fell under lower evidence category. Conversely, a study from Spain reported that 86% of the IVIg prescribed were for labelled indications, 10% were for off-label but scientifically supported indications, and only 4% were for off-label unsupported indications [11]. This suggests rational use of IVIg is possible with strict adherence to the labelled indications and diagnostic criteria.

IVIg recorded the highest single-drug expenditure in the studied period. The total cost of IVIg use was RM 695,426.36, noting that this was partially subsidized by the National Blood Centre. The actual unsubsidized projected cost of IVIg may exceed RM 900,000, a substantial amount which could be used for other therapeutic causes. Meanwhile, 38.5% of the total cost was spent on indications in lower evidence category and this imposed a huge cost burden on the hospital. Meanwhile, a previous study in Saudi Arabia by Alangari and colleagues revealed that IVIg was used as off-label or non-recommended indications among two-third of the population, incurring a cost of $431,325. [12]. The exorbitant costs of off-label and non-recommended IVIg use warrants evidence-based prescribing in clinical practice.

Although the use of IVIg treatment for septicaemia in immunocompromised patient is an approved indication in the MOHM Formulary, it has mixed evidence. Due to weak evidence of efficacy from previous studies, International Guidelines for Management of Sepsis and Septic Shock recommended against the use of IVIg in sepsis [13]. Jarczak and colleagues argued that IVIg appeared to be a safe treatment option for sepsis and septic shock, but further clinical data is required to assess the cost–benefit ratio [14]. Thus, the use of IVIg for septicaemia in immunocompromised patients should be constantly reviewed and evaluated based on current guidelines and evidence.

While the use of IVIg in treating Kawasaki disease was classified as high evidence according to American Academy of Allergy, Asthma and immunology [9], its use was not indicated in the local formulary. Timely review of IVIg use policy and development of national IVIg prescribing protocol based on updated evidence may provide guidance and standardization of IVIg usage across nationwide health facilities in the future.

In our study, the use of IVIg in nine SLE cases had costed RM 113,000. However, the use of IVIg in SLE was classified as lower evidence category. A reduction of the SLE disease activity index score suggested the beneficial effects of IVIg therapy [15]. IVIg was found to be beneficial in specific clinical manifestations of SLE including nephritis, cerebral lupus and serositis [16]. Hence, the use of IVIg in SLE should be decided on a case by case basis, prioritized based on clinical manifestations with proven efficacies.

Despite its high cost, IVIg played important role when no other cost-effective alternative available. For instance, the usage of IVIg in primary immunodeficiency with hypogammaglobulinemia may reduce mortality and morbidity, along with improved quality of life [17]. Meanwhile, the use of high-dose IVIg in ITP was established more than two decades ago [18]. Nevertheless, a recent review of recent evidence showed that eltrombopag, a non-peptide thrombopoietin appeared to be more cost-effective than IVIg treatment [19]. Similarly, newer options such as subcutaneous immunoglobulin and corticosteroids was found to be comparable to IVIg which was once known to be the only effective agent in treating CIDP [20]. A timely review of latest evidence is, therefore, warranted to inform and update current IVIg prescribing guidelines.

There were several limitations in this study. First, there was several medical records that could not be traced, and the actual usage could be higher than that reported. Second, records of outpatient IVIg use were not collected. As IVIg is often administered on an outpatient basis, future research should include this setting. Third, the small sample size has attributed to the short study duration. A longer study period with a larger sample size would improve generalizability of findings. Furthermore, we did not measure the clinical outcome of each case and hence calculation of incremental cost-effectiveness (ICER) ratio was not performed. This should be considered in future studies.

## Conclusions

The majority of IVIg in this study was prescribed for off-label use. A timely revision of IVIg use policy, as well as the establishment of a national IVIg prescribing guideline may provide standardization in its usage and encourage evidence-based prescribing of IVIg.

## Supplementary Information

**Additional file 1: Table S1.** IVIg approved indications based on MOHM, FDA, EMA and AAAAI evidence categories. **Table S2.** Definition on evidence category and strength of recommendation. **Table S3.** Total expenditures for indications in which evidence for its use was not established

## Data Availability

The data sets generated and/or analyzed during the current study are not publicly available due to confidentiality of patients, but are available from the corresponding author on reasonable request.
