# Supplementary material for: Intravenous human immunoglobulin utilization patterns and cost analysis in a Malaysian tertiary referral hospital
Source: J Pharm Policy Pract. 2022 Apr 26;15:31. doi: 10.1186/s40545-022-00430-2 (PMC9040375; doi:10.1186/s40545-022-00430-2)
Supplement: Supplementary file 1 — Additional file 1: Table S1. IVIg approved indications based on MOHM, FDA, EMA and AAAAI evidence categories. Table S2. Definition on evidence category and strength of recommendation. Table S3. Total expenditures for indications in which evidence for its use was not established [file 40545_2022_430_MOESM1_ESM.docx]

**Table S1: IVIg approved indications based on MOHM, FDA, EMA and AAAAI evidence categories**

| **Indications** | **MOH** | **FDA** | **EMA** | **AAAAI Evidence category** |
| --- | --- | --- | --- | --- |
| Primary immunodeficiency with hypogammaglobulinemia and impaired specific antibody production | **✓** | **✓** | **✓** | **Ia** |
| Secondary immunodeficiency due to CLL | **✓** | **✓** | **✓** | **Ib** |
| Idiopathic thrombocytopenia purpura | **✓** | **✓** | **✓** | **Ia** |
| Kawasaki syndrome | **-** | **✓** | **✓** | **Ia** |
| Gullain-Barre Syndrome | **✓** | **-** | **✓** | **Ib** |
| Multifocal motor neuropathy | **-** | **✓** | **✓** | **Ib** |
| Chronic inflammatory demyelinating polyneuropathy | **-** | **✓** | **✓** | **Ia** |
| Multifocal motor neuropathy | **-** | **✓** | **✓** | **Ib** |
| Chronic inflammatory demyelinating polyneuropathy | **-** | **✓** | **✓** | **Ia** |
| Pediatric human immunodeficiency  virus infection | **-** | **✓** | **-** | **Ib** |
| Prevention of graft versus host disease and infection in adult bone marrow transplantation | **-** | **✓** | **-** | **Ib** |
| Septicemia in immunocompromised patients or  patients not responding to antibiotics | **✓** | **-** | **-** | **NA** |

**Table S2: Definition on evidence category and strength of recommendation**

| **Category** | **Definition** |
| --- | --- |
| **Evidence category** |  |
| **Ia** | From meta-analysis of RCTs |
| **Ib** | From at least one RCT |
| **IIa** | From at least one controlled trial without randomization |
| **IIb** | From at least one other type of quasi-experimental study |
| **III** | From non-experimental descriptive studies such as comparative, correlation or case-control studies |
| **IV** | From expert committee reports or opinions or clinical experience of respected authorities or both |
| **Strength of recommendations** |  |
| **A** | Based on category I evidence |
| **B** | Based on category II evidence or extrapolated from category I evidence |
| **C** | Based on category III evidence or extrapolated from category I or II evidence |
| **D** | Based on category IV evidence or extrapolated from category I, II or III evidence |

**Table S3: Total expenditures for indications in which evidence for its use was not established**

| **Indication** | **N** | **Total cost (RM)** |
| --- | --- | --- |
| Septicaemia in immunocompromised patients | 4 | 35,062.00 |
| Neonate of mother with immune thrombocytopenia | 2 | 1,492.00 |
| Sensomotor peripheral neuropathy | 1 | 14,920.00 |
| Myelodysplastic syndrome | 1 | 3,730.00 |
| Chronic myelogenous leukemia | 1 | 5,222.00 |
| Multiple myeloma | 1 | 1,119.00 |
| Antiphospholipid syndrome | 1 | 8,952.00 |
| Viral myocarditis | 1 | 1,119.00 |
| **Total** | 12 | 71,616.00 |
